# Dynamic predictive scores for cardiac surgery-associated agitated delirium: a single-center retrospective observational study

**DOI:** 10.1186/s13019-023-02339-6

**Published:** 2023-07-06

**Authors:** Yu Tian, Bingyang Ji, Xiaolin Diao, Chunrong Wang, Weiwei Wang, Yuchen Gao, Sudena Wang, Chun Zhou, Qiaoni Zhang, Sizhe Gao, Xinyi Xu, Jia Liu, Jianhui Wang, Yuefu Wang

**Affiliations:** 1 Department of Anesthesiology, Beijing AnZhen Hospital, Capital Medical University, Beijing, China; 2 Department of Cardiopulmonary Bypass, Fuwai Hospital,National Centre for Cardiovascular Diseases, Chinese Academy of Medical Sciences and Peking Union Medical College, Beijing, China; 3 Department of Information Center, Fuwai Hospital, National Centre for Cardiovascular Diseases, Chinese Academy of Medical Sciences and Peking Union Medical College, Beijing, China; 4 Department of Anesthesiology, Fuwai Hospital, National Centre for Cardiovascular Diseases, Chinese Academy of Medical Sciences and Peking Union Medical College, Beilishi Road 167, Xicheng District, Beijing, 100037 China; 5 Department of Surgery Intensive Care Unit, Beijing Shijitan Hospital, Capital Medical University, No. 10 Tieyi Road, Yangfangdian, Haidian District, Beijing, China

**Keywords:** Adult cardiac surgery, Agitated delirium, Risk prediction score systems

## Abstract

**Background:**

Prevention, screening, and early treatment are the aims of postoperative delirium management. The scoring system is an objective and effective tool to stratify potential delirium risk for patients undergoing cardiac surgery.

**Methods:**

Patients who underwent cardiac surgery between January 1, 2012, and January 1, 2019, were enrolled in our retrospective study. The patients were divided into a derivation cohort (n = 45,744) and a validation cohort (n = 11,436). The AD predictive systems were formulated using multivariate logistic regression analysis at three time points: preoperation, ICU admittance, and 24 h after ICU admittance.

**Results:**

The prevalence of AD after cardiac surgery in the whole cohort was 3.6% (2,085/57,180). The dynamic scoring system included preoperative LVEF ≤ 45%, serum creatinine > 100 µmol/L, emergency surgery, coronary artery disease, hemorrhage volume > 600 mL, intraoperative platelet or plasma use, and postoperative LVEF ≤ 45%. The area under the receiver operating characteristic curve (AUC) values for AD prediction were 0.68 (preoperative), 0.74 (on the day of ICU admission), and 0.75 (postoperative). The Hosmer‒Lemeshow test indicated that the calibration of the preoperative prediction model was poor (*P* = 0.01), whereas that of the pre- and intraoperative prediction model (*P* = 0.49) and the pre, intra- and postoperative prediction model (*P* = 0.35) was good.

**Conclusions:**

Using perioperative data, we developed a dynamic scoring system for predicting the risk of AD following cardiac surgery. The dynamic scoring system may improve the early recognition of and the interventions for AD.

**Supplementary Information:**

The online version contains supplementary material available at 10.1186/s13019-023-02339-6.

## Introduction

Delirium is an acute confusional state affecting consciousness, attention, cognition, and perception [1]. Patients who undergo cardiac surgery are at high risk for delirium because they are more likely to be older and often have multiple comorbidities, such as hypertension and diabetes [2]. It has been reported that the prevalence of delirium after cardiac surgery varies widely (4–51%) [3–5]. The wide range could be explained by the heterogeneity of the patient population, timing of the assessments, hospital location, delirium subtype, and selected assessment tool. Postoperative delirium, especially after cardiac surgery, can foreshadow poor outcomes [6]. From the patient^’^s view, delirium and subsequent sequelae, including cognitive decline, loss of independence, increased costs, and increased mortality, are among the most feared adverse events following cardiac surgery [7]. It has been estimated that approximately $6.9 billion (in American dollars) of Medicare hospital expenditures are attributable to delirium [8]. Thus, it is increasingly important for clinicians to have an understanding of postoperative delirium.

Clinically, delirium can be divided into hyperactive, hypoactive, and mixed types [9]. Agitated delirium (AD), a hyperactive subset, is a disease state characterized by changes in mental status combined with psychomotor agitation, metabolic derangements, and hyperthermia [10]. Hypoactive delirium is a disease state characterized by somnolence and silence. Mixed types have some clinical features of both AD and hypoactive delirium. Until now, the definition of AD has been derived primarily from clinical criteria, and the diagnosis tends to be subjective [11]. In 2019, Rood [12] indicated that 7% of intensive care unit (ICU) delirium patients were hyperactive, 36% were mixed type, and 27% were hypoactive. Patients with delirium frequently present with hypoactivity or mixed type; however, the hyperactive subtype is easily recognized. Based on past studies, the cause of delirium is multifactorial, and there are many risk factors that predispose patients to delirium, including older age, dementia (often not recognized clinically), functional disabilities, and the complex interactions of comorbidities [13]. To date, many studies have reported that the symptoms, etiology, pathophysiology, detection rates, treatment experience and outcomes of different subtypes of delirium are highly heterogeneous [14]. Consequently, the focus has been on identifying clinically meaningful subtypes.

Prevention, screening, and early treatment are the aims of postoperative delirium management. Although the reported risk factors vary and are unlikely to change, identification of patients with these factors can allow clinicians to direct preventive efforts toward at-risk patients. Delirium remains underdiagnosed in the perioperative setting, but screening and assessment tools are readily available to aid clinicians in identifying delirium. Prediction tools also allow the patient and their family to be better informed about risks. Delirium may be prevented or attenuated when risk stratification tools are used. The purpose of this study was to derive and validate a dynamic scoring system that could predict the risk of AD following cardiac surgery during the preoperative and early postoperative periods.

## Methods

### Patients

The research was approved by the Ethics Committee of Fuwai Hospital (20191308), and the requirement for written informed consent was waived for all patients. Patients were included if they were older than 18 years of age and had undergone open cardiac surgery (including coronary artery bypass grafting (CABG), valve surgery, great vessel operations, congenital heart disease repair, cardiac tumor surgery and combined surgery). The exclusion criteria were adult patients with previous renal replacement therapy or dialysis (n = 70), epilepsy (n = 152), shock (n = 899), a cardiac assist device (n = 5), delirium (n = 0), septicemia (n = 1), sleep apnea syndrome (n = 138), sleep disorder (n = 15), or pulmonary embolism (n = 25). A total of 1289 patients were excluded. Finally, the charts of 57,180 patients were enrolled retrospectively (Additional file 1: Fig. S1).

### Data collection

All digital clinical data were provided by the electronic records at the Fuwai Hospital Information Center. We monitored and then checked the collected perioperative data. The data were checked twice by postgraduates and engineers who work for the Hospital Information Center. A total of 31 candidate variables were directly extracted from the patient medical charts according to previous literature [17–26]. We examined the interactions between the risk variables, and none were clinically or statistically significant. The definitions of risk variables were mainly referenced from the STS website [https://www.sts.org/registries/sts-national-database].

### Diagnosis of agitated delirium

Based on the STS definition, AD is defined as short-term mental disturbances marked by illness, confusion, and cerebral excitement or the need for medical intervention with medication such as olanzapine and droperidol. The diagnosis of AD was limited to 24 h after ICU admission. Postgraduates and engineers collected the data from review charts and diagnose agitated delirium. They looked for clinical manifestations and diagnostic criteria of AD within the patient medical records and a history of medical intervention with medication such as olanzapine and droperidol. They were primarily responsible for deciding the AD diagnosis after searching and reviewing the clinical charts after the data were checked twice. The Confusion Assessment Method for the Intensive Care Unit (CAM-ICU) is the best for diagnosing AD but was not available in the ICU at our institute for the majority of patients, but the agitated subtype was documented by the nursing staff. Furthermore, in the general ward, the treatment of AD with olanzapine and droperidol was determined by clinical personnel in accordance with the results of the mini-mental test, the confusion assessment method and other relevant evidence of AD and then recorded in the medical chart. The diagnosis of AD requires both medication records and nursing records.

### Statistical analysis

Statistical analysis was performed with SPSS software for Windows (version 25.0. IBM Corp, Armonk, NY). We transformed continuous variables into categorical variables according to clinically meaningful cutoff values or values reported in previous literature^3^. Categorical variables are expressed as a frequency (n) and percentage (%) and were analyzed using Pearson’s chi-square test or Fisher’s exact test as appropriate. When less than 2% of the values were missing for a variable, single imputation was used (defaulted to the most common value of the variable), whereas if more than 2% of the values were missing, the missing values were modeled as unknown.

The data set was randomly divided into a derivation cohort (n = 45,744) and a validation cohort (n = 11,436) by SPSS software (the ratio was 4:1). The rationale for using these variables in the scoring model was based on the results of univariate analyses and the clinical relevance of the variables. AD predictive systems were formulated using multivariate logistic regression analysis at three time points: preoperation, ICU admittance, and 24 h after ICU admittance. The calibration of the prediction models was assessed by the Hosmer–Lemeshow goodness-of-fit test, and the discriminatory ability of the models was assessed by the area under the receiver operating characteristic (ROC) curve (AUC). The final prediction scores were the nearest integer to the regression coefficient. The AUC and Hosmer–Lemeshow goodness-of-fit test were applied to estimate the reliability of the prediction scoring systems in the validation cohort.

## Results

In this retrospective study, we included 58,469 adult patients who underwent cardiac repair at our institute between January 1, 2012, and January 1, 2019. A total of 57,180 patients (45,744 patients in the derivation cohort and 11,436 in the validation cohort) treated during the 7-year period were retrospectively analyzed. The prevalence of AD after cardiac surgery in the whole cohort was 3.6% (2085/57180), whereas in the derivation and validation cohorts, it was 3.3% (1504/45744) and 5.1% (581/11436), respectively. The mortality rate in the whole cohort was 0.9% (504/57180), whereas in the AD cohort, it was 1.4% (29/2085). The baseline clinical characteristics of patients in the derivation and validation cohorts are illustrated in Additional file 1: Table S1. AD was associated with higher medical costs and a longer hospital stay (*P* < 0.001, Additional file 1: Table S2). AD was also associated with mortality (*P* = 0.011). However, postoperative AD was not correlated with the risk of pulmonary complications or reintubation requirement of (*P* = 0.572, *P* = 0.496, Additional file 1: Table S3).

The perioperative information of the patients in the derivation group is shown in Table 1.

**Table 1:** Clinical characteristics of derivation cohort (N = 45,744) (%)

Preoperative variable	No agitated delirium (n = 44240, 96.7%)	Agitated delirium (n = 1504, 3.3%)	*P* Value
Age (year)			< 0.001
< 60	25,056 (56.6)	728 (48.4)	
60–74	17,246 (39.0)	693 (46.1)	
≥ 75	1938 (4.4)	83 (5.5)	
Gender			< 0.001
Female	15,460 (34.9)	307 (20.4)	
Male	28,780 (65.1)	1197 (79.6)	
Obesity (BMI ≥ 30 kg/m^2^)	3454 (7.8)	158 (10.5)	< 0.001
Previous COPD	631 (1.4)	37 (2.5)	0.001
History of cardiac surgery	1145 (2.6)	31 (2.1)	0.204
IABP use	43 (0.1)	4 (0.3)	0.068
Infective endocarditis	338 (0.8)	14 (0.9)	0.467
Proteinuria	630 (1.4)	37 (2.5)	0.001
Hypertension	14,916 (33.7)	592 (39.4)	< 0.001
Type 2 diabetes	8543 (19.3)	327 (21.7)	0.019
NYHA classification = 4	1087 (2.5)	76 (5.1)	< 0.001
LVEF			< 0.001
Normal (> 60%)	43,347 (98.0)	1436 (95.5)	
Mild damage (46%-60%)	695 (1.6)	45 (3.0)	
Moderate damage (30–45%)	178 (0.4)	20 (1.3)	
Severe damage (< 30%)	20 (0.0)	3 (0.2)	
Serum creatinine (umol/L)			< 0.001
< 70	14,547 (32.9)	298 (19.8)	
70–100	25,164 (56.9)	904 (60.1)	
101–120	3059 (6.9)	172 (11.4)	
121–150	895 (2.0)	60 (4.0)	
> 150	575 (1.3)	70 (4.7)	
Emergency surgery	1817 (4.1)	217 (14.4)	< 0.001
Alcohol user	35,829 (81.0)	1296 (86.2)	< 0.001
Tobacco exposure			< 0.001
Yes	19,388 (43.8)	802 (53.3)	
Unknown	1094 (2.5)	17 (1.1)	
Carotid artery stenosis	1187 (2.7)	62 (4.1)	0.001
History of stroke	2993 (6.8)	136 (9.0)	0.001
Coronary artery disease	42,651 (96.4)	1481 (98.5)	< 0.001
Albumin (g/L)			< 0.001
Normal (35–45)	27,271 (61.6)	994 (66.1)	
Abnormal (< 35 OR > 45)	16,628 (37.6)	487 (32.4)	
Unknown	341 (0.8)	23(1.5)	
Total protein (g/L)			< 0.001
> 65	33,790 (76.4)	1021 (67.9)	
≤ 65	10,111 (22.9)	460 (30.6)	
Unknown	339 (0.8)	23 (1.5)	
Simvastatin use	15,405 (34.8)	505 (33.6)	0.319
Beta-blocker use	23,717 (53.6)	830 (55.2)	0.228
The type of surgery			< 0.001
Others*	3107 (7.0)	91 (6.1)	
CABG	19,988 (45.2)	673 (44.7)	
Valve Surgery	10,159 (23.0)	317 (21.1)	
Great vessel surgery	3374 (7.6)	159 (10.6)	
Combined surgery	7612 (17.2)	264 (17.6)	
*Intraoperative variable*			
Bypass time (min)			0.008
Off-pump surgery	12,036 (27.2)	393 (26.1)	
≤ 120 min	22,399 (50.6)	727 (48.3)	
> 120 min	9805 (22.2)	384 (25.5)	
Hemorrhage volume			< 0.001
≤ 600 mL	37,606 (85.0)	1003 (66.7)	
> 600 mL	6634 (15.0)	501 (33.3)	
RBC use	7322 (16.6)	434 (28.9)	< 0.001
Platelet use	2187 (4.9)	270 (18.0)	< 0.001
Plasma use	4744 (10.7)	406 (27.0)	< 0.001
*Postoperative variable*			
LVEF (> 60%,reference group)	11,655 (26.3)	328 (21.8)	< 0.001
Mild damage (46%-60%)	16,959 (38.3)	667 (44.3)	
Moderate damage (30–45%)	2616 (5.9)	163 (10.8)	
Severe damage (< 30%)	131 (0.3)	11 (0.7)	
Unknown	12,879 (29.1)	335 (22.3)	
Serum creatinine (umol/L)			< 0.001
< 70	13,624 (30.8)	364 (24.2)	
70–100	21,243 (48.0)	674 (44.8)	
101–120	3253 (7.4)	161 (10.7)	
121–150	909 (2.1)	77 (5.1)	
> 150	252 (0.6)	36 (2.4)	
Unknown	4959 (11.2)	192 (12.8)	

Data presented as numbers and percentages

*BMI* body mass index; *COPD* chronic obstructive pulmonary disease; *IABP* Intra-aortic ballon pump; *CABG* coronary artery bypass grafting; *LVEF* left ventricular ejection fraction; *NYHA* New York Heart Association; *RBC* red blood cell

*included congenital heart disease repair, cardiac tumor surgery

### Analysis of the risk variables

The details of the preoperative prediction model for AD are shown in Additional file 1: Table S4, whereas the pre- and intraoperative prediction model is shown in Additional file 1: Table S5, and the pre, intra- and postoperative prediction model is shown in Additional file 1: Table S6. The risk variables contributing to AD were age, male sex, obesity, previous chronic obstructive pulmonary disease (COPD), hypertension, type 2 diabetes, New York Heart Association (NYHA) classification = 4, low preoperative left ventricular ejection fraction (LVEF), elevated serum creatinine, emergency surgery, alcohol use, carotid artery stenosis, history of stroke, coronary artery disease, low total protein, type of surgery, intraoperative hemorrhage volume > 600 mL, intraoperative red blood cell (RBC) count, platelet or plasma use and low postoperative LVEF.

### Diagnostic utility of the prediction score

1. AD prediction model based on preoperative variables

**Fig. 1:**
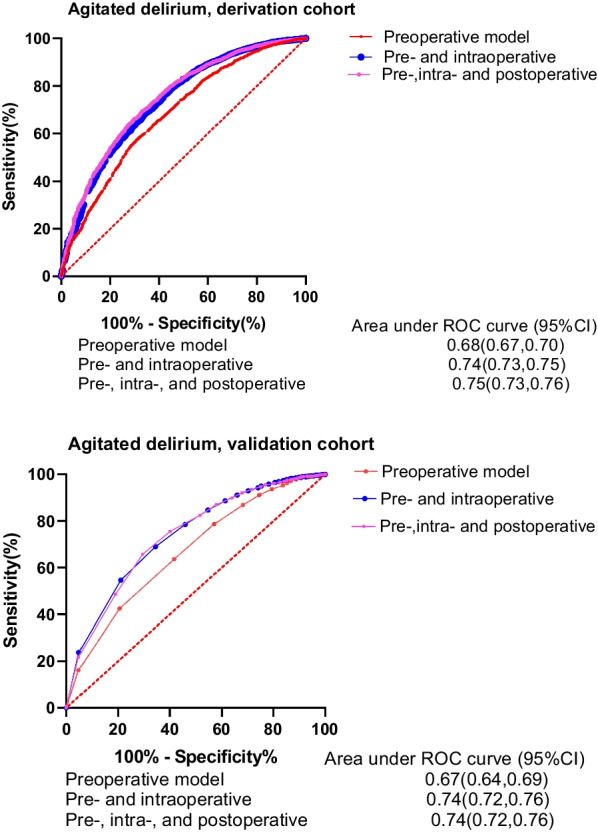
Receiver operating characteristic curves (ROCs) for the AD prediction models in the derivation cohort and validation cohort

After using only the preoperative variables in the derivation cohort to construct the AD prediction model, the AUC for AD was 0.68 (95% CI, 0.67, 0.70, Fig. 1), and in the validation cohort, the AUC was 0.67 (95% CI, 0.64, 0.69, Fig. 1). Nevertheless, the calibration according to the Hosmer‒Lemeshow test was poor for this model (P = 0.01). The sensitivity, specificity, positive predictive value, and negative predicted value for predicting the medium- and high-risk groups were 37.5%, 83.1%, 10.6%, and 96.1%, respectively.

2. AD prediction model based on pre- and intraoperative variables

In the derivation data set, the performance of the AD risk prediction model, which was based on pre- and intraoperative variables, was as follows: AUC = 0.74 (95% CI, 0.73, 0.75, Fig. 1), indicating good discrimination ability. It was very similar to the validation cohort (AUC = 0.74, 95% CI, 0.72, 0.76). The Hosmer‒Lemeshow test showed that the calibration of the prediction model was good (*P* = 0.49). The sensitivity, specificity, positive predictive value, and negative predicted value for predicting the medium- and high-risk groups were 49.7%, 82.2%, 13.00%, and 96.80%, respectively.

3. AD prediction model based on pre, intra, and postoperative variables

The AUC for the AD prediction model with the derivation cohort was 0.75 (95% CI, 0.73, 0.76, Fig. 1). The Hosmer‒Lemeshow test demonstrated good calibration for the derivation cohort (*P* = 0.35). The performance with the validation cohort also indicated good discriminability (AUC = 0.74, 95% CI, 0.72, 0.76). The sensitivity, specificity, positive predictive value, and negative predicted value for predicting the medium- and high-risk groups were 52.7%, 79.5%, 12.10%, and 96.90%, respectively.

### Development of the prediction score

**Fig. 2:**
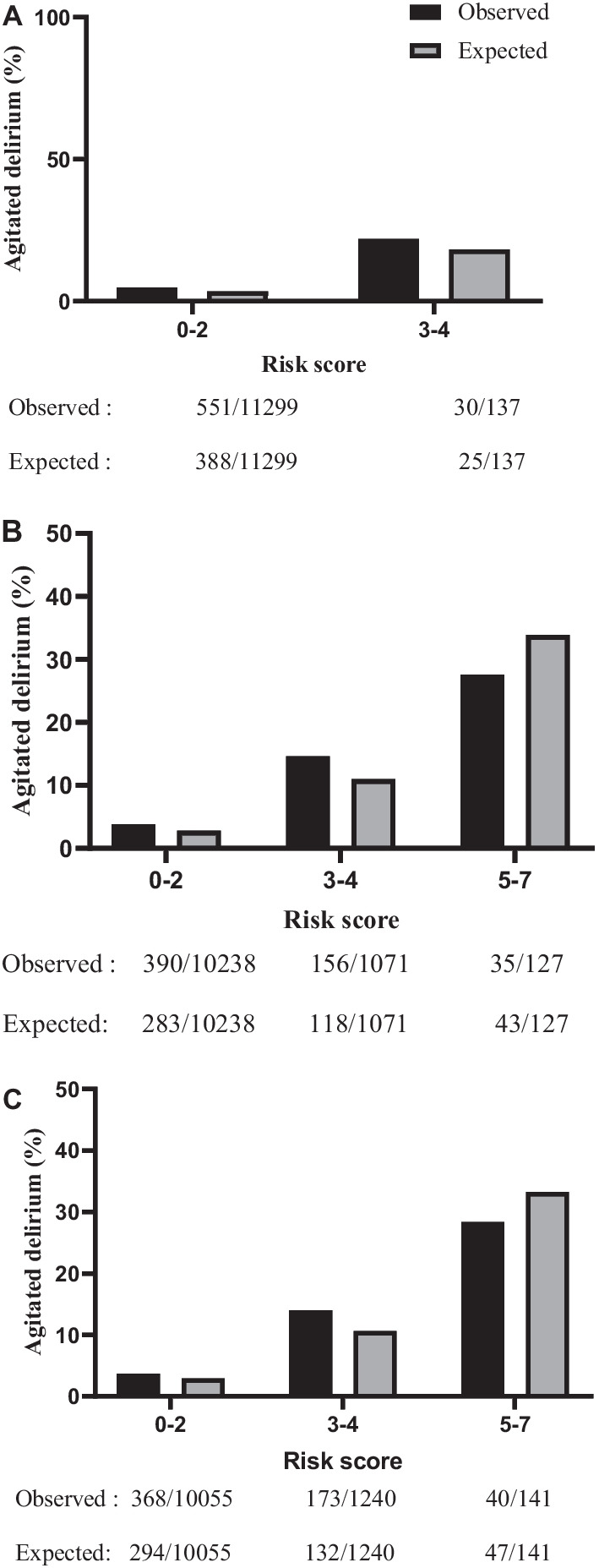
Observed and predicted risk of agitated delirium in the validation cohorts. **A** preoperative, **B** at the day of ICU admittance, **C** at 24 h after ICU admittance

Three scoring systems for predicting postoperative AD after cardiac surgery are presented in Table 2. The prediction scoring systems were developed from regression coefficients of data from the derivative cohort patients. Depending on the scoring system, the predicted risks of AD could be grouped into three classifications: low, medium, and high, in accordance with the practically observed incidence of AD (Table 3). In the validation cohorts, the incidences of AD onset predicted by the model were similar to those observed clinically (Fig. 2). The risk scores and their associated predictive risks are presented in Additional file 1: Table S7.

**Table 2:** Prediction scores for postoperative agitated delirium after cardiac surgery

Preoperative scores	Scores
LVEF ≤ 45%	1
Serum creatinine > 100umol/L	1
Emergency surgery	1
Coronary artery disease	1
*Pre- and intraoperative scores*	
LVEF ≤ 45%	1
Serum creatinine > 100umol/L	1
Emergency surgery	1
Coronary artery disease	1
Hemorrhage volume > 600 ml	1
Intraoperative platelet use	1
Intraoperative plasma use	1
*Pre-, intra- and postoperative scores*	
Serum creatinine > 100umol/L	1
Emergency surgery	1
Coronary artery disease	1
Hemorrhage volume > 600 ml	1
Intraoperative platelet use	1
Intraoperative plasma use	1
Postoperative LVEF ≤ 45%	1

*LVEF* left ventricular ejection fraction

**Table 3:** Risk Stratification of agitated delirium after cardiac surgery at indicated time points

Total score	Preoperative	ICU admittance	24 h after ICU admittance
0–2	Low (< 5.0%)	Low (< 5.0%)	Low (< 5.0%)
3–4	Medium–high (≥ 5.0%)	Medium (5.0–25%)	Medium (5.0–25%)
5–7		High (> 25%)	High (> 25%)

Example: if a patient get total scores ≤ 2, his predicted risk of postoperative agitated delirium following cardiac surgery is low (less than 5.0%)

## Discussion

Our study developed dynamic scoring systems for predicting the risk of AD following cardiac surgery. The dynamic scoring systems evolved from the perioperative risk variables of a large cohort of cardiac patients, and they can predict the risk of AD following cardiac surgery during the preoperative and early postoperative periods. These dynamic scoring systems were also well validated internally. Additionally, the rationale for exclusion was critically ill patients and the frequency of each variable. Although some of these criteria (e.g., shock, dialysis, sepsis) would increase the risk of delirium, the frequency of those variables for each group was too small.

Delirium, a postoperative complication affecting brain function, can severely affect the quality of life of patients, especially in the aging population. Similar to previous investigations^2^, we found that postoperative delirium was associated with negative outcomes. Several authors agree that early detection could prevent or assist in the treatment of delirium [15]. Some of the proposed preventive interventions have been shown to have beneficial effects on delirium, such as early mobilization, early extubation and minimization of alarm use and noisiness. Patients undergoing cardiac surgery are at a higher risk of developing postoperative delirium due to several factors, including surgical complexity, comorbidities and age [16]. On the basis of the prediction model, we created a simple bedside dynamic scoring card that can be used to proactively identify the risk strata (low, medium, or high risk) for AD at three time points (preoperation, ICU admittance, and 24 h after ICU admittance).

Our research represents a meaningful attempt to prove that an elevated preoperative serum creatinine level is a risk factor for AD following cardiac surgery. The risk of AD for patients whose preoperative serum creatinine was more than 150 µmol/L was nearly two times higher than that of those whose serum creatinine was less than 70 µmol/L. Katznelson et al. [17] found that the estimated odds ratio for patients who had preoperative creatinine levels greater than 150 µmol/L was 2.96 (95% CI 1.90–4.63, P < 0.001), which was similar to our study results. In 2013, Koster et al. [18] indicated that the European System for Cardiac Operative Risk Evaluation (EuroSCORE) score predicted the risk of delirium following cardiac surgery, and the EuroSCORE includes creatinine concentrations greater than 200 µmol/L as a risk variable (http://www.euroscore.org/calcold.html). In 2017, Siew et al. [19] found that the daily peak serum creatinine value was a risk variable for delirium (OR = 1.35, 95% CI, 1.18–1.55) and coma (OR = 1.44, 95% CI, 1.20–1.74) during critical illness. In 2020, Mossello et al.[20] indicated that moderate renal impairment was independently associated with delirium among older fracture patients aged 75–84. The association between serum creatinine and delirium requires further analysis [21, 22].

Prediction scores in previous investigations were derived from information collected from preoperative variables [17, 18]. We included intraoperative and postoperative predictors in the prediction score and derived dynamic predictive scores for cardiac surgery-associated AD. Moreover, we found that ICU admittance (or after surgery) was the best time point at which to employ the prediction model. The AUC of the pre- and intraoperative prediction model was better than that of the preoperative prediction model (0.74 vs. 0.68) and was similar to that of the pre, intra- and postoperative prediction model (0.74 vs. 0.75). Compared with the investigations carried out by Tse et al. [23] (n = 679) and Mufti et al. [24] (n = 5,584), our study has an unprecedentedly larger sample size. Other authors also attempted to develop a predictive model for delirium through a prospective study: Katznelson et al. [17] in 2009 (n = 1,059), Koster et al. [18] in 2013 (n = 300), Krzych et al. [3] in 2014 (n = 5,781), and Kumar et al. [25] in 2017 (n = 120). However, those investigations were based on a small sample size and were not internally validated. Although the preoperative prediction rule for delirium after cardiac surgery developed by Rudolph [26] in 2009 was internally validated (AUC in the derivation cohort was 0.74; AUC in the validation cohort was 0.75), it had a tendency to be imprecise, outdated and even eventually eliminated. Given the differences in delirium definition and race, no statistical comparison can be performed among the abovementioned models.

As in most hospital wards [27], the majority of patients in our institute are not routinely monitored for delirium, but the presence of the agitated subtype is recorded in medical charts. The incidence of AD was low, only 3.6%. The reason was that we only included the agitated subtype of delirium, which represents only a small proportion of all delirium cases [9]. This limits the ability to generalize the dynamic scoring systems to types of delirium other than the agitated subtype. Our study is a retrospective analysis, and the majority of patients in our institute are not routinely monitored for delirium; hence, we can only identify the hyperactive subtype. Postoperative serum creatinine and LVEF were usually measured within 24 h of ICU admission in our institution. Hence, the postoperative variable can be measured prior to the onset of delirium. It may be difficult to perform in other institutions. However, our study can help to further explore effective clinical interventions for delirium and reduce the associated adverse effects.

There were several limitations to our study. First, as a retrospective analysis that is pending prospective validation, our observation data were mainly based on chart extraction. The quality of the acquired data might profoundly impact the results and their interpretation, although the data were checked twice by postgraduates and engineers. Additionally, in our study, the postoperative prediction model could not predict AD within 24 h of ICU admission. Delirium typically peaks at 24–72 h, and no patients were found to have AD within 24 h according to our results. We thought 24 h after ICU admission was a good time point for reassessment. Furthermore, it can be difficult to compare the results of this study with those of other studies because the definition of delirium and the method to diagnose delirium are quite different. Finally, data were obtained from a single center, and the scoring system needs to be validated in other centers.

## Conclusion

Using perioperative data, we developed a dynamic scoring system for AD following cardiac surgery that can be used to flag patient risk. Dynamic scoring systems may improve the early recognition of and the interventions for AD. This scorecard will contribute to software development and further explorations into effective clinical interventions for delirium.

## Supplementary Information

**Additional file 1.** Details of this study which include table and figure.

## Data Availability

The data sets used and/or analyzed during the current study are available from the corresponding author on reasonable request.

## Abbreviations

AD: Agitated delirium
ICU: Intensive care unit
CABG: Coronary artery bypass grafting
CAM-ICU: Confusion assessment method for the intensive care unit
ROC: Receiver operating characteristic
AUC: Area under the receiver operating characteristic curve
COPD: Chronic obstructive pulmonary disease
IABP: Intra-aortic balloon pump
NYHA: New York Heart Association
RBC: Red blood cell
LVEF: Left ventricular ejection fraction
OR: Odds ratio
CI: Confidence intervals
BMI: Body mass index
IQR: Interquartile range
