# Supplementary material for: Dynamic predictive scores for cardiac surgery-associated agitated delirium: a single-center retrospective observational study
Source: J Cardiothorac Surg. 2023 Jul 6;18:219. doi: 10.1186/s13019-023-02339-6 (PMC10327302; doi:10.1186/s13019-023-02339-6)

| **eTable 1. Clinical Characteristics of Derivation and Validation Cohort(%)** | | |
| --- | --- | --- |
| **Preoperative variable** | **Derivation Cohort (N=45744)** | **Validation Cohort（N=11436）** |
| Age（year） |  |  |
| <60 | 25784(56.4) | 6417(56.1) |
| 60-74 | 17939(39.2) | 4652(40.7) |
| ≥75 | 2021(4.4) | 367(3.2) |
| Gender |  |  |
| Female | 15767(34.5) | 3911(34.2) |
| Male | 29977(65.5) | 7525(65.8) |
| [Obesity（BMI≥30](javascript:;)[kg/m²）](javascript:;) | 3612(7.9) | 643(5.6) |
| Previous COPD | 668(1.5) | 126(1.1) |
| History of cardiac surgery | 1176(2.6) | 281(2.5) |
| IABP use | 47(0.1) | 9(0.1) |
| Infective endocarditis | 352(0.8) | 65(0.6) |
| Proteinuria | 667(1.5) | 219(1.9) |
| Hypertension | 15508(33.9) | 3530(30.9) |
| Type 2 diabetes | 8870(19.4) | 2297(20.1) |
| NYHA classification=4 | 1163(2.5） | 236(2.1) |
| LVEF(>60%,reference group) | 44783(97.9) | 10773(94.2) |
| Mild damage(46%-60%) | 740(1.6) | 553(4.8) |
| Moderate damage(30-45%) | 198(0.4) | 97(0.8) |
| Severe damage(<30%) | 23(0.1) | 13(0.1) |
| Serum creatinine(umol/L) |  |  |
| <70 | 14845(32.5) | 3221(28.2) |
| 70-100 | 26068(57.0) | 6693(58.5) |
| 101-120 | 3231(7.1) | 1020(8.9) |
| 121-150 | 955(2.1) | 325(2.8) |
| >150 | 645(1.4) | 177(1.5) |
| Emergency surgery | 2034(4.4) | 514(4.5) |
| Alcohol user | 37125(81.2) | 9736(85.1) |
| Tobacco exposure |  |  |
| Yes | 20190(44.1) | 4926(43.1) |
| Unknown | 1111(2.4) | 255(2.2) |
| Carotid artery stenosis | 1249(2.7) | 427(3.7) |
| History of stroke | 3129(6.8) | 932(8.1) |
| Coronary artery disease | 44132(96.5) | 10953(95.8) |
| Albumin(g/L) |  |  |
| Normal(35-45) | 28265(61.8) | 7737(67.7) |
| Abnormal(<35 OR >45) | 17115(37.4) | 3612(31.6) |
| Unknown | 364(0.8) | 87(0.8) |
| Total protein (g/L) |  |  |
| >65 | 34811(76.1) | 8656(75.7) |
| ≤65 | 10571(23.1) | 2686(23.5) |
| Unknown | 362(0.8) | 94(0.8) |
| Simvastatin use | 15910(34.8) | 3781(33.1) |
| Beta-blocker use | 24547(53.7) | 5994(52.4) |
| The type of surgery |  |  |
| Others* | 3198(7.0) | 613(5.4) |
| CABG | 20661(45.2) | 4812(42.1) |
| Valve Surgery | 10476(22.9) | 2413(21.1) |
| Great vessel surgery | 3533(7.7) | 964(8.4) |
| Combined surgery | 7876(17.2) | 2634(23.0) |
| **Intraoperative variable** |  |  |
| Bypass time |  |  |
| Off-pump surgery | 12429(27.2) | 3029(26.5) |
| ≤120min | 23126(50.6) | 5449(47.6) |
| >120min | 10189(22.3) | 2958(25.9) |
| Hemorrhage volume |  |  |
| ≤600mL | 38609(84.4) | 8776(76.7) |
| ＞600mL | 7135(15.6) | 2660(23.3) |
| RBC use | 7756(17.0) | 2102(18.4) |
| Platelet use | 2457(5.4) | 676(5.9) |
| Plasma use | 5150(11.3) | 955(8.4) |
| **Postoperative variable** |  |  |
| LVEF(>60%,reference group) | 11983(26.2) | 3046(26.6) |
| Mild damage(46%-60%) | 17626(38.5) | 5281(46.2) |
| Moderate damage(30-45%) | 2779(6.1) | 627(5.5) |
| Severe damage(<30%) | 142(0.3) | 34(0.3) |
| Unknown | 13214(28.9) | 2448(21.4) |
| Serum creatinine(umol/L) |  |  |
| <70 | 13988(30.6) | 3472(30.4) |
| 70-100 | 21917(47.9) | 5620(49.1) |
| 101-120 | 3414(7.5) | 929(8.1) |
| 121-150 | 986(2.2) | 292(2.6) |
| >150 | 288(0.6) | 111(1.0) |
| Unknown | 5151(11.3) | 1012(8.8) |
| Agitated delirium | 1504(3.3) | 581(5.1) |
| Data presented as numbers and percentages. BMI, body mass index; CABG, coronary artery bypass grafting; LVEF, left ventricular ejection fraction;COPD, chronic obstructive pulmonary disease;IABP,Intra-aortic ballon pump;NYHA, New York Heart Association; RBC, red blood cell.*included congenital heart disease repair , cardiac tumor surgery. | | |

| **eTable 2. Length of stay and total cost following cardiac surgery by agitated delirium, data from the whole cohort (N=57180).** | | | | | |
| --- | --- | --- | --- | --- | --- |
|  |  | **Total cost(yuan),P<0.001** | | **Length of stay, P<0.001** | |
| **Agitated delirium** | **Number(%）** | **Median** | **IQR** | **Median** | **IQR** |
| None | 55095(96.4) | 91442 | (77212,115226) | 13 | (10,18) |
| YES | 2085(3.6) | 137599 | (102536,193124) | 16 | (12,22) |
| IQR, interquartile range | | | | | |

| **eTable 3: Outcomes following cardiac surgery by agitated delirium, data from the whole cohort(N=57180).** | | | | | | |
| --- | --- | --- | --- | --- | --- | --- |
| **Agitated delirium** | **Pulmonary complication*** | | **re-intubation** | | **Died in hospital** | |
|  | **Number**  **(%)** | **Odds ratio**  **(95% CI)** | **Number**  **(%)** | **Odds ratio**  **(95% CI)** | **Number**  **(%)** | **Odds ratio**  **(95% CI)** |
| None | 2987(5.4) | 1 | 673(1.2) | 1 | 475(0.9) | 1 |
| YES | 119(5.7) | 1.056(0.874,1.275) | 22(1.1) | 0.862(0.563,1.322) | 29(1.4) | 1.622(1.112,2.366) |
| P Value | 0.572 |  | 0.496 |  | 0.011 |  |
| CI, Confidence interval; *Pulmonary complication included pneumonia, pulmonary edema, pulmonary atelectasis and pulmonary embolism. | | | | | | |

| **eTable 4. Preoperative prediction model for postoperative agitated delirium after cardiac surgery.** | | | | | |
| --- | --- | --- | --- | --- | --- |
| **Preoperative** | **Coefficient** | **Scores** | **OR** | **95%CI** | **P value** |
| Age（<60 years, reference group) |  |  |  |  | <0.001 |
| 60-74 | 0.346 | 0 | 1.414 | (1.263, 1.582） | <0.001 |
| ≥75 | 0.247 | 0 | 1.409 | (1.109,1.790) | 0.005 |
| Male | 0.470 | 0 | 1.599 | (1.363, 1.876） | <0.001 |
| [Obesity（BMI≥30kg/m²）](javascript:;) | 0.350 | 0 | 1.419 | (1.195, 1.685） | <0.001 |
| Previous COPD | 0.365 | 0 | 0.038 | (1.441, 1.021） | 0.038 |
| Hypertension | 0.185 | 0 | 1.203 | (1.073,1.348) | 0.002 |
| Type 2 diabetes | 0.137 | 0 | 1.146 | (1.007,1.305) | 0.038 |
| NYHA classification=4 | 0.474 | 0 | 1.607 | (1.251,2.064) | <0.001 |
| LVEF(>60%,reference group) |  |  |  |  | <0.001 |
| Mild damage(46%-60%) | 0.480 | 0 | 1.616 | (1.176,2.222) | 0.003 |
| Moderate damage(30-45%) | 0.896 | 1 | 2.450 | (1.503,3.994) | <0.001 |
| Severe damage(<30%) | 1.248 | 1 | 3.483 | (0.972,12.486) | 0.055 |
| Serum creatinine (<70umol/L,reference group) | |  |  |  |  |
| 70-100 | 0.328 | 0 | 1.388 | (1.199,1.608) | <0.001 |
| 101-120 | 0.693 | 1 | 2.001 | (1.629,2.458) | <0.001 |
| 121-150 | 0.789 | 1 | 2.201 | (1.634,2.963) | <0.001 |
| >150 | 1.315 | 1 | 3.726 | (2.787,4.983) | <0.001 |
| Emergency surgery | 1.168 | 1 | 3.216 | (2.738,3.778) | <0.001 |
| Alcohol user | 0.426 | 0 | 1.531 | (1.315,1.783) | <0.001 |
| Carotid artery stenosis | 0.301 | 0 | 1.351 | (1.033,1.766) | 0.028 |
| History of stroke | 0.270 | 0 | 1.310 | (1.089,1.576) | 0.004 |
| Coronary artery disease | 0.727 | 1 | 2.069 | (1.361,3.144) | 0.001 |
| Total protein (>65g/L,reference group) | |  |  |  | <0.001 |
| ≤65 | 0.292 | 0 | 1.339 | (1.193,1.501) | <0.001 |
| Unknown | 0.176 | 0 | 1.192 | (0.758,1.873) | 0.446 |
| The type of surgery(Others*,reference group) | |  |  |  | 0.002 |
| CABG | -0.165 | 0 | 0.848 | (0.669,1.074) | 0.171 |
| Valve Surgery | -0.093 | 0 | 0.911 | (0.716,1.160) | 0.451 |
| Great vessel surgery | 0.186 | 0 | 1.204 | (0.920,1.576) | 0.177 |
| Combined surgery | 0.014 | 0 | 1.014 | (0.791,1.300) | 0.911 |
| Intercept | -5.549 |  |  |  | <0.001 |
| Minimum score = 0; maximum score =4. BMI, body mass index; COPD, chronic obstructive pulmonary disease; CABG, coronary artery bypass grafting; CI, confidence interval; LVEF, left ventricular ejection fraction; OR, odds ratio; RBC, red blood cell.*included congenital heart disease repair , cardiac tumor surgery. | | | | | |

| **eTable 5. Pre- and intraoperative prediction model for postoperative agitated delirium after cardiac surgery.** | | | | | |
| --- | --- | --- | --- | --- | --- |
| **Preoperative** | **Coefficient** | **Scores** | **OR** | **95%CI** | **P value** |
| Age（<60 years, reference group) |  |  |  |  | <0.001 |
| 60-74 | 0.273 | 0 | 1.315 | (1.176, 1.469) | <0.001 |
| ≥75 | 0.247 | 0 | 1.281 | (1.007,1.629) | 0.044 |
| Male | 0.452 | 0 | 1.571 | (1.361, 1.815） | <0.001 |
| [Obesity（BMI≥30kg/m²）](javascript:;) | 0.346 | 0 | 1.413 | (1.188, 1.682） | <0.001 |
| Previous COPD | 0.406 | 0 | 1.501 | (1.061, 2.125） | 0.022 |
| Hypertension | 0.119 | 0 | 1.127 | (1.009,1.258) | 0.035 |
| Type 2 diabetes | 0.214 | 0 | 1.239 | (1.086,1.412) | 0.001 |
| NYHA classification=4 | 0.356 | 0 | 1.427 | (1.107,1.840) | 0.006 |
| LVEF(>60%,reference group) |  |  |  |  | <0.001 |
| Mild damage(46%-60%) | 0.435 | 0 | 1.546 | (1.122,2.130) | 0.008 |
| Moderate damage(30-45%) | 0.861 | 1 | 2.364 | (1.450,3.856) | 0.001 |
| Severe damage(<30%) | 1.019 | 1 | 2.771 | (0.767,10.005) | 0.120 |
| Serum creatinine (<70umol/L,reference group) | |  |  |  |  |
| 70-100 | 0.292 | 0 | 1.339 | (1.155,1.553) | <0.001 |
| 101-120 | 0.591 | 1 | 1.806 | (1.466,2.225) | <0.001 |
| 121-150 | 0.614 | 1 | 1.849 | (1.364,2.506) | <0.001 |
| >150 | 1.153 | 1 | 3.167 | (2.353,4.262) | <0.001 |
| Emergency surgery | 0.879 | 1 | 2.408 | (2.027,2.860) | <0.001 |
| Alcohol user | 0.352 | 0 | 1.422 | (1.219,1.658) | <0.001 |
| Carotid artery stenosis | 0.358 | 0 | 1.430 | (1.092,1.872) | 0.009 |
| History of stroke | 0.269 | 0 | 1.309 | (1.085,1.577) | 0.005 |
| Coronary artery disease | 0.741 | 1 | 2.098 | (1.378,3.194) | 0.001 |
| Total protein (>65g/L,reference group) | |  |  |  | <0.001 |
| ≤65 | 0.267 | 0 | 1.306 | (1.163,1.467) | <0.001 |
| Unknown | 0.441 | 0 | 1.554 | (0.989,2.442) | 0.056 |
| Hemorrhage volume＞600mL | 0.800 | 1 | 2.227 | (1.982,2.501) | <0.001 |
| Intraoperative RBC use | 0.456 | 0 | 1.577 | (1.395,1.784) | <0.001 |
| Intraoperative platelet use | 0.616 | 1 | 1.852 | (1.557,2.203) | <0.001 |
| Intraoperative plasma use | 0.546 | 1 | 1.726 | (1.501,1.984) | <0.001 |
| Intercept | -5.910 |  |  |  | <0.001 |
| Minimum score = 0; maximum score = 7. BMI, body mass index; COPD, chronic obstructive pulmonary disease; CI, confidence interval; LVEF, left ventricular ejection fraction; OR, odds ratio; RBC, red blood cell.*included congenital heart disease repair , cardiac tumor surgery. | | | | | |

| **eTable 6. Pre-,intra- and postoperative prediction model for postoperative agitated delirium after cardiac surgery.** | | | | | |
| --- | --- | --- | --- | --- | --- |
| **Preoperative variable** | **Coefficient** | **Scores** | **OR** | **95%CI** | **P value** |
| Age（<60 years, reference group) |  |  |  |  | <0.001 |
| 60-74 | 0.267 | 0 | 1.306 | (1.169, 1.460) | <0.001 |
| ≥75 | 0.250 | 0 | 1.284 | (1.009,1.635) | 0.042 |
| Male | 0.435 | 0 | 1.545 | (1.335, 1.788） | <0.001 |
| [Obesity（BMI≥30kg/m²）](javascript:;) | 0.352 | 0 | 1.421 | (1.194, 1.691） | <0.001 |
| Previous COPD | 0.418 | 0 | 1.518 | (1.072, 2.150） | 0.019 |
| Hypertension | 0.127 | 0 | 1.135 | (1.016,1.268) | 0.025 |
| Type 2 diabetes | 0.193 | 0 | 1.213 | (1.063,1.385) | 0.004 |
| NYHA classification=4 | 0.317 | 0 | 1.373 | (1.063,1.773) | 0.015 |
| Serum creatinine (<70umol/L,reference group) | |  |  |  |  |
| 70-100 | 0.319 | 0 | 1.376 | (1.178,1.608) | <0.001 |
| 101-120 | 0.542 | 1 | 1.719 | (1.365,2.165) | <0.001 |
| 121-150 | 0.476 | 1 | 1.610 | (1.152,2.251) | 0.005 |
| >150 | 1.070 | 1 | 2.916 | (2.078,4.090) | <0.001 |
| Emergency surgery | 0.940 | 1 | 2.559 | (2.151,3.044) | <0.001 |
| Alcohol user | 0.352 | 0 | 1.422 | (1.220,1.659) | <0.001 |
| Carotid artery stenosis | 0.369 | 0 | 1.446 | (1.104,1.894) | 0.007 |
| History of stroke | 0.280 | 0 | 1.323 | (1.097,1.596) | 0.003 |
| Coronary artery disease | 0.754 | 1 | 2.126 | (1.397,3.237) | <0.001 |
| Total protein (>65g/L,reference group) | |  |  |  | <0.001 |
| ≤65 | 0.263 | 0 | 1.301 | (1.158,1.462) | <0.001 |
| Unknown | 0.482 | 0 | 1.619 | (1.033,2.536) | 0.035 |
| **Intraoperative variable** |  |  |  |  |  |
| Hemorrhage volume＞600ml | 0.770 | 1 | 2.160 | (1.921,2.429) | <0.001 |
| Intraoperative RBC use | 0.462 | 0 | 1.588 | (1.403,1.796) | <0.001 |
| Intraoperative platelet use | 0.642 | 1 | 1.900 | (1.596,2.261) | <0.001 |
| Intraoperative plasma use | 0.553 | 1 | 1.738 | (1.512,1.998) | <0.001 |
| **Postoperative variable** |  |  |  |  |  |
| LVEF(>60%,reference group) |  |  |  |  | <0.001 |
| Mild damage(46%-60%) | 0.264 | 0 | 1.302 | (1.134,1.495) | <0.001 |
| Moderate damage(30-45%) | 0.609 | 1 | 1.839 | (1.503,2.250) | <0.001 |
| Severe damage(<30%) | 0.645 | 1 | 1.905 | (0.989,3.672) | 0.054 |
| Unknown | -0.122 | 0 | 0.885 | (0.755,1.039) | 0.135 |
| Intercept | -5.936 |  |  |  | <0.001 |
| Minimum score = 0; maximum score = 7. BMI, body mass index; COPD, chronic obstructive pulmonary disease; CI, confidence interval; LVEF, left ventricular ejection fraction; OR, odds ratio; RBC, red blood cell.*included congenital heart disease repair , cardiac tumor surgery. | | | | | |

| **eTable 7: Risk of agitated delirium associated total score** | |
| --- | --- |
| **Total point** | **Estimated risk of agitated delirium associated with total score** |
| **Preoperative** |  |
| 0 | 0.0038763 |
| 1 | 0.0104671 |
| 2 | 0.0279497 |
| 3 | 0.0724937 |
| 4 | 0.1752307 |
| **ICU Admittance** |  |
| 0 | 0.0027049 |
| 1 | 0.0073185 |
| 2 | 0.0196468 |
| 3 | 0.0516614 |
| 4 | 0.1289809 |
| 5 | 0.2869998 |
| 6 | 0.5224848 |
| 7 | 0.7483817 |
| **24 Hours after ICU Admittance** |  |
| 0 | 0.0026356 |
| 1 | 0.0071320 |
| 2 | 0.0191522 |
| 3 | 0.0504024 |
| 4 | 0.1260880 |
| 5 | 0.2817090 |
| 6 | 0.5159945 |
| 7 | 0.7434542 |

**eFigure 1: the study patient flow diagram.**


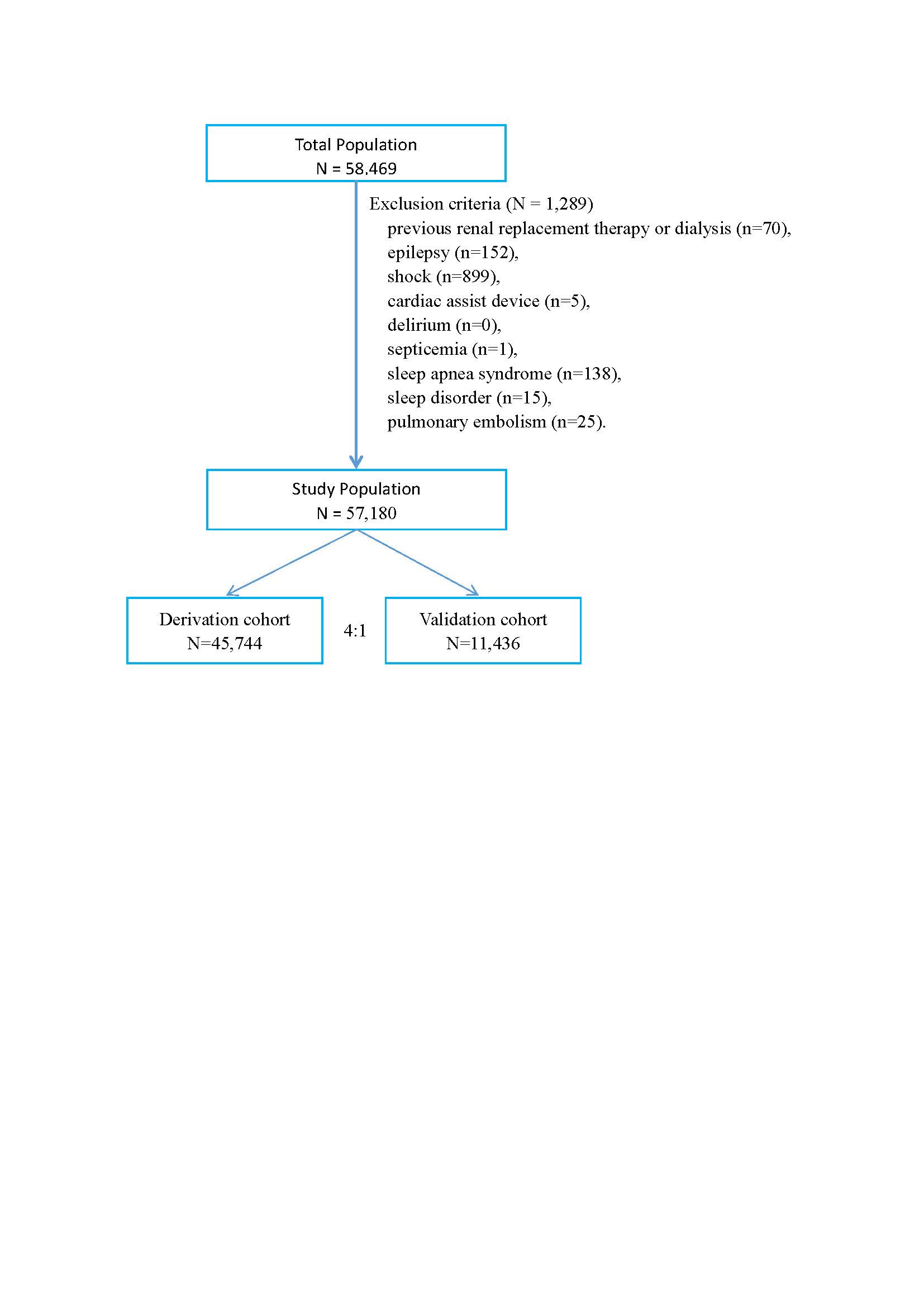

Supplement: Supplementary file 1 — Additional file 1. Details of this study which include table and figure. [file 13019_2023_2339_MOESM1_ESM.docx]
